# A Comparative Study on Pediatric Diabetic Ketoacidosis Before and During the COVID-19 Pandemic in a Tertiary Care Centre in South India

**DOI:** 10.7759/cureus.116704

**Published:** 2026-09-22

**Authors:** Thannoli Gowthami Gowrinathan, Rupa Devi P, Ravichandran Revathy, Nehaashri K, Poovazhagi Varadarajan, Anuradha D

**Affiliations:** 1 Paediatrics and Child Health, Madras Medical College, Chennai, IND; 2 Pediatrics, Government Peripheral Hospital, Perambur, Chennai, IND; 3 General Practice, Madras Medical College, Chennai, IND; 4 Paediatrics, Vels Medical College and Hospital, Periyapalayam, IND

**Keywords:** clinical presentation of dka, covid-19, covid and dka, dka outcome, pediatric dka

## Abstract

Introduction

Diabetic ketoacidosis (DKA) is a serious acute complication of type 1 diabetes mellitus in children. The COVID-19 pandemic disrupted healthcare access and diabetes care, potentially increasing the severity of pediatric DKA. This study compared the clinical profile, biochemical characteristics, complications and outcomes of pediatric DKA before and during the COVID-19 pandemic and evaluated the association of COVID-19 immunoglobulin G (IgG) status with disease severity and outcome.

Study

This hospital-based comparative cohort study was conducted at the Institute of Child Health and Hospital for Children, Egmore, Chennai. Children aged one month to 12 years admitted with DKA during 2019 (pre-COVID, n=58) and 2021 (COVID era, n=78) were included. Demographic, clinical, biochemical and outcome parameters were compared. During 2021, SARS-CoV-2 IgG antibody status was also assessed.

Results

A total of 136 children were studied. The mean age and duration of hospital and ICU stay were comparable between the groups. Admission blood glucose was significantly higher in 2019 (482.6±112.9 vs 433.0±121.2 mg/dL; p=0.016), whereas glycatd hemoglobin (HbA1c) was significantly higher in 2021 (11.83±3.15 vs 10.81±2.49%; p=0.044). Sepsis was significantly more frequent during the COVID era (35.1% vs 15.5%; p=0.011). Decreased urine output was also significantly more frequent in 2021. Acute kidney injury, cerebral oedema, mechanical ventilation and poor outcomes were numerically higher during the COVID period but did not reach statistical significance. COVID-19 IgG positivity was not significantly associated with DKA severity, complications or outcome. Severe metabolic acidosis, renal dysfunction, hypernatremia and major complications were strongly associated with poor outcome.

Conclusion

The COVID-19 pandemic was associated with poorer glycaemic control and increased sepsis among children presenting with DKA. Previous SARS-CoV-2 exposure did not independently influence disease severity or outcome. Early recognition, prompt treatment and uninterrupted diabetes care are essential to reduce adverse outcomes.

## Introduction

Diabetic ketoacidosis (DKA) is the most serious acute complication of type 1 diabetes mellitus (T1DM) in children and remains an important cause of diabetes-related morbidity and mortality worldwide [1]. It results from absolute or relative insulin deficiency, leading to hyperglycaemia, ketosis and metabolic acidosis [2]. Despite advances in the diagnosis and management of childhood diabetes, DKA continues to be the initial presentation in a substantial proportion of newly diagnosed children and may also occur in those with established diabetes because of missed insulin doses, intercurrent illness or delayed medical attention [3]. Severe DKA may result in life-threatening complications including cerebral oedema, acute kidney injury, shock and multiorgan dysfunction if not recognized and treated promptly [4].

The coronavirus disease 2019 (COVID-19) pandemic profoundly affected healthcare delivery and health-seeking behaviour across the world. Delayed access to medical care, lockdown restrictions, fear of hospital visits and disruption of routine healthcare services were recognized during the pandemic and contributed to delayed presentation of children with acute illnesses [5]. Several international studies subsequently reported an increase in new-onset T1DM and/or DKA among children during the COVID-19 era [6]. Studies from Germany, Korea and Saudi Arabia similarly reported changes in the incidence and clinical characteristics of pediatric DKA during the pandemic [7]. A subsequent meta-analysis of observational studies also demonstrated an overall increase in pediatric DKA during the COVID-19 pandemic compared with the pre-pandemic period [8].

The mechanisms underlying these observations remain incompletely understood. Direct effects of SARS-CoV-2 infection on glucose metabolism and pancreatic β-cell function have been proposed, although the contribution of direct viral effects compared with indirect effects related to healthcare disruption and delayed presentation remains uncertain [9]. Data from the Indian pediatric population are comparatively limited, with previous Indian studies primarily describing the clinical profile and outcomes of pediatric DKA [10]. South Indian studies have additionally highlighted the importance of infection and systemic complications as determinants of outcome in children with DKA [11].

Understanding whether the COVID-19 pandemic altered the clinical profile, laboratory parameters, complications and outcomes of pediatric DKA is therefore important for improving early diagnosis and optimizing management strategies. Therefore, the present study aimed to compare the clinical characteristics, biochemical parameters, complications and outcomes of children with DKA before and during the COVID-19 pandemic and to evaluate factors associated with poor outcomes. The primary objective of this study was to compare the clinical presentation, biochemical parameters, severity, complications and outcomes of pediatric diabetic ketoacidosis before the COVID-19 pandemic (2019) and during the COVID-19 pandemic (2021). The secondary objective was to evaluate the association between SARS-CoV-2 IgG antibody status and clinical presentation, disease severity, complications, and outcomes among children with diabetic ketoacidosis in the 2021 cohort.

## Materials and methods

This hospital-based comparative cohort study was conducted in the Department of Pediatric Diabetology at the Institute of Child Health and Hospital for Children, Egmore, Chennai, South India. The study was designed to compare the clinical profile, biochemical characteristics, complications and outcomes of pediatric DKA before and during the COVID-19 pandemic. Ethical approval was obtained from the Institutional Ethics Committee before the commencement of the study, and the study adhered to the principles of the Declaration of Helsinki. Written informed consent was obtained from parents or legal guardians for the prospective component and for COVID-19 antibody testing.

The study included children aged one month to 12 years admitted with a diagnosis of DKA. DKA was diagnosed according to the International Society for Pediatric and Adolescent Diabetes (ISPAD) clinical practice consensus guidelines, requiring all three biochemical criteria: hyperglycaemia (blood glucose >200 mg/dL (11 mmol/L)), metabolic acidosis (venous pH <7.3 and/or serum bicarbonate <18 mmol/L) and ketonaemia or ketonuria [12]. Children with incomplete medical records or alternative causes of metabolic acidosis were excluded.

The study population comprised two cohorts. The pre-COVID cohort included children admitted between January 2019 and December 2019 whose data were collected retrospectively from hospital records. The COVID-era cohort included children admitted between January 2021 and December 2021 who were enrolled prospectively. This design enabled a comparison between periods before and during the pandemic while maintaining uniform diagnostic and treatment protocols at the same tertiary care centre.

For every participant, demographic variables including age, sex, nutritional status, duration of symptoms and history of previously diagnosed diabetes were recorded. Clinical presentation at admission included polyuria, polydipsia, vomiting, abdominal pain, fever, respiratory distress, altered sensorium, seizures, dehydration, urine output and haemodynamic status. New-onset diabetes presenting as DKA was identified separately from known cases of type 1 diabetes.

Laboratory investigations included random blood glucose, glycated haemoglobin (HbA1c), arterial or venous blood gas analysis, serum bicarbonate, serum electrolytes, blood urea nitrogen, serum creatinine and ketone estimation. DKA severity was classified as mild, moderate or severe according to accepted ISPAD criteria based on venous pH and serum bicarbonate values. Shock was defined as documented circulatory compromise with age-specific hypotension and/or clinical signs of poor peripheral perfusion (capillary refill time >3 seconds, weak/absent peripheral pulses, cold extremities or altered sensorium), with requirement for fluid resuscitation and/or vasoactive support during the hospital stay [12]. Acute kidney injury (AKI) was defined as an acute deterioration in renal function, evidenced by an increase in serum creatinine and/or a clinically significant reduction in urine output during hospitalization [12]. Sepsis was defined as suspected or confirmed infection associated with systemic clinical features and evidence of organ dysfunction, as documented by the treating clinical team [12]. Cerebral edema was defined as clinically suspected cerebral swelling occurring during diabetic ketoacidosis, characterized by acute neurological deterioration such as altered sensorium, severe headache, recurrent vomiting, abnormal neurological findings, or other clinical features suggestive of cerebral edema [12]. All children with DKA were managed according to standard pediatric DKA treatment protocols, including assessment of hydration and hemodynamic status, appropriate isotonic fluid resuscitation followed by controlled fluid replacement, intravenous regular insulin infusion, serial monitoring and correction of blood glucose and electrolytes, addition of dextrose-containing fluids as required during insulin therapy, monitoring for and management of complications including cerebral edema and transition to subcutaneous insulin following resolution of ketoacidosis [12].

For children recruited during the COVID-19 period, SARS-CoV-2 IgG antibodies were assessed using the Abbott ARCHITECT SARS-CoV-2 IgG chemiluminescent microparticle immunoassay (CMIA) (Abbott Laboratories, Abbott Park, IL, USA), which detects IgG antibodies against the SARS-CoV-2 nucleocapsid protein. The results were reported as a signal-to-cutoff (S/CO) ratio, with a ratio of ≥1.4 interpreted as positive and a ratio of <1.4 interpreted as negative, according to the manufacturer’s criteria. Patients were categorised into antibody-positive and antibody-negative groups, and subgroup analyses were undertaken to determine whether previous COVID-19 exposure influenced clinical presentation, disease severity, complications or outcomes.

Primary outcome measures included DKA severity at presentation, duration of hospital stay, need for intensive care, mechanical ventilation, inotropic support and in-hospital outcome. Secondary outcomes included acute kidney injury, cerebral oedema, shock, respiratory distress and other major complications. The frequency of newly diagnosed diabetes presenting with DKA during the two study periods was also analysed. For outcome analysis, survival to hospital discharge was considered a good outcome, whereas in-hospital death was considered a poor outcome.

Data were entered into Microsoft Excel (Microsoft, Redmond, WA) and analysed using IBM SPSS Statistics version 26 (IBM Corp, Armonk, NY). Continuous variables were tested for normality using Shapiro-Wilk test and expressed as mean±standard deviation or median with interquartile range as appropriate. Categorical variables were presented as frequencies and percentages. Comparisons between groups were performed using the independent Student's t-test or Mann-Whitney U test for continuous variables and Chi-square test or Fisher's exact test for categorical variables. Variables included in the analysis were assessed for completeness before statistical analysis. Because the study included all eligible DKA admissions during the predefined study periods, an a priori sample-size calculation was not performed; the sample size was determined by the number of eligible admissions available during those periods. Analyses were performed using the available observations for each variable, and no imputation of missing values was undertaken. For continuous variables, Cohen's d was calculated as the standardized mean difference between the 2019 and 2021 groups. The sign of Cohen's d indicates the direction of the difference according to the order of group subtraction, while the absolute value represents the magnitude of the standardized effect. Factors associated with poor outcome were evaluated using univariate analyses. Given the limited number of poor-outcome events, multivariable logistic regression was not used for definitive estimation of independent predictors because of the potential risk of model overfitting. A two-sided p-value of less than 0.05 was considered statistically significant.

## Results

A total of 136 children diagnosed with DKA were included in the study. Fifty-eight children were admitted during the pre-COVID period (2019), while 78 children were admitted during the COVID-19 period (2021). Clinical characteristics, laboratory parameters, complications and outcomes were compared between the two cohorts. The mean age was comparable between the pre-COVID and COVID-era cohorts (7.47±3.24 years vs 7.98±3.21 years, respectively; p=0.364). Similarly, the mean duration of hospital stay (10.8±5.52 vs 9.89±5.56 days; p=0.346) and ICU stay (1.38±1.60 vs 1.84±1.94 days; p=0.148) did not differ significantly between the two groups. Serum urea (34.38±19.25 vs 42.07±36.23 mg/dL; p=0.144) and creatinine (0.79±0.41 vs 1.04±0.97 mg/dL; p=0.068) were numerically higher during the COVID period, although these differences were not statistically significant. Admission blood glucose was significantly higher in the pre-COVID cohort compared with the COVID-era cohort (482.61±112.95 vs 433.02±121.17 mg/dL; p=0.016), whereas HbA1c was significantly higher during the COVID period (11.83±3.15% vs 10.81±2.49%; p=0.044). Overall, the two cohorts were comparable with respect to most demographic and laboratory parameters, with statistically significant differences observed only in admission blood glucose and HbA1c (Table 1).

**Table 1:** Comparison of demographic and laboratory parameters *p<0.05 (Statistically significant). SD: Standard deviation; mg/dL: milligram per decilitre; t: independent t-test; df: degree of freedom; HbA1c: glycated hemoglobin.

Variable	2019 (Mean±SD)	2021 (Mean±SD)	t value	df	p value	Cohen's d (effect size)
Age (years)	7.47±3.24	7.98±3.21	-0.913	134	0.363	0.159
Hospital stay (days)	10.80±5.52	9.89±5.56	0.953	134	0.342	0.165
ICU stay (days)	1.38±1.60	1.84±1.94	-1.485	134	0.140	0.256
Urea (mg/dL)	34.38±19.25	42.07±36.23	-1.438	134	0.153	0.250
Creatinine (mg/dL)	0.79±0.41	1.04±0.97	-1.822	134	0.071	0.316
Blood sugar (mg/dL)	482.61±112.95	433.02±121.17	2.435	134	0.016*	0.412
HbA1c (%)	10.81±2.49	11.83±3.15	-2.034	134	0.044*	0.361

The clinical characteristics, complications and outcomes of children with DKA in the 2019 and 2021 cohorts were compared. Polyuria was present in 36 (62.1%) children in 2019 and 50 (64.1%) children in 2021 (p=0.808). Polydipsia was reported in 33 (56.9%) and 47 (60.3%) children, respectively (p=0.694). Respiratory distress was observed in 31 (53.4%) children in 2019 and 42 (53.8%) children in 2021 (p=0.963). Decreased urine output was observed in five (6.4%) children in 2021 compared with none in 2019 (p=0.049). AKI was present in 15 (25.9%) children in 2019 and 25 (32.1%) children in 2021 (p=0.433). Mechanical ventilation was required in eight (13.8%) children in 2019 and 15 (19.2%) children in 2021 (p=0.403). Cerebral oedema occurred in 13 (22.4%) children in 2019 and 24 (30.8%) children in 2021 (p=0.279). Sepsis was documented in nine (15.5%) children in 2019 and 27 (35.1%) children in 2021 (p=0.011). Poor outcome occurred in five (8.6%) children in 2019 and 12 (15.4%) children in 2021 (p=0.238) (Table 2).

**Table 2:** Comparison of clinical characteristics and outcomes n: number of the study population; *p<0.05 (Statistically significant); φ: phi-effect size; df: degree of freedom.

Variable	n (%) (2019)	n (%) (2021)	p value	χ² value	df	Effect size (φ)
Polyuria	36 (62.1%)	50 (64.1%)	0.808	0.059	1	0.021
Polydipsia	33 (56.9%)	47 (60.3%)	0.694	0.155	1	0.034
Respiratory distress	31 (53.4%)	42 (53.8%)	0.963	0.002	1	0.004
Decreased urine output	0 (0.0%)	5 (6.4%)	0.049*	3.860	1	0.168
AKI	15 (25.9%)	25 (32.1%)	0.433	0.614	1	0.067
Mechanical ventilation	8 (13.8%)	15 (19.2%)	0.403	0.700	1	0.072
Cerebral oedema	13 (22.4%)	24 (30.8%)	0.279	1.173	1	0.093
Sepsis	9 (15.5%)	27 (34.6%)	0.011*	6.234	1	0.214
Poor outcome	5 (8.6%)	12 (15.4%)	0.238	1.391	1	0.101

Among children in the COVID-era cohort, comparison according to COVID-19 IgG antibody status showed no statistically significant differences in age, hospital stay, ICU stay, blood glucose or HbA1c. The mean age was 8.2±3.4 years in the COVID IgG-positive group and 7.8±3.1 years in the IgG-negative group (p=0.543). Mean hospital stay was 9±7 days and 10±4 days, respectively (p=0.333), while mean ICU stay was 2±2 days in both groups (p=0.642). Mean blood glucose was 425±102 mg/dL in the IgG-positive group and 440±136 mg/dL in the IgG-negative group (p=0.583), and mean HbA1c was 12.1±3.3% and 11.6±3.0%, respectively (p=0.544). The mean COVID-19 antibody titre was significantly higher in the IgG-positive group (49.77 ± 43.65) compared with the IgG-negative group (2.06±2.14; p<0.001) (Table 3).

**Table 3:** Comparison of continuous variables according to COVID-19 IgG antibody status IgG: Immunoglobullin G; SD: standard deviation; IU/ml: internarional Units per millilitre; mg/dL: milligram per decilitre; df: degree of freedom; t value: derived from independent t-test.

Variable	COVID IgG Positive (Mean±SD)	COVID IgG Negative (Mean±SD)	p value	t value	df	Effect size (Cohen’s d)
Age (in years)	8.2±3.4	7.8±3.1	0.543	0.543	76	0.123
Hospital stay (days)	9±7	10±4	0.333	-0.788	76	-0.179
ICU stay (days)	2±2	2±2	0.642	0.000	76	0.000
Blood sugar (mg/dL)	425±102	440±136	0.583	-0.543	76	-0.123
HbA1c (%)	12.1±3.3	11.6±3	0.544	0.701	76	0.159
COVID antibody titre (IU/mL)	49.77±43.65	2.06±2.14	<0.001	7.081	76	1.608

Among the overall study population, comparison according to clinical outcome showed significant differences in several laboratory parameters. The mean hospital stay was 11±5 days in children with good outcome and 4±4 days in those with poor outcome (p<0.001), while mean ICU stay was 1±2 days and 3±3 days, respectively (p=0.009). Mean serum urea was 33±18 mg/dL in the good-outcome group compared with 76±60 mg/dL in the poor-outcome group (p<0.001), and mean serum creatinine was 0.8±0.4 mg/dL and 2.1±1.6 mg/dL, respectively (p<0.001). Mean pH was 7.124 in the good-outcome group and 6.919 in the poor-outcome group (p<0.001), while mean serum bicarbonate was 7.9 mmol/L and 4.4 mmol/L, respectively (p=0.001). Mean serum sodium was 133 mmol/L in the good-outcome group and 139 mmol/L in the poor-outcome group (p<0.001). Mean serum potassium was 4.59 mmol/L and 4.04 mmol/L, respectively (p=0.013) (Table 4).

**Table 4:** Comparison of laboratory parameters according to overall outcome ICU: Intensive Care Unit; pH: power of hydrogen; SD: standard deviation; t: independent t test; df: degree of freedom.

Variable	Good outcome (Mean±SD)	Poor outcome (Mean±SD)	p value	t value	df	Effect size (Cohen’s d)
Hospital stay (days)	11±5	4±4	<0.001	5.519	134	1.431
ICU stay (days)	1±2	3±3	0.009	-3.598	134	-0.933
Urea (mg/dL)	33±18	76±60	<0.001	-6.201	134	-1.608
Creatinine (mg/dL)	0.8±0.4	2.1±1.6	<0.001	-7.503	134	-1.945
pH	7.124±0.12	6.919±0.12	<0.001	6.589	134	1.708
Bicarbonate (mg/dL)	7.9±3.6	4.4±3.6	0.001	3.750	134	0.972
Sodium (meq/L)	133±5.7	139±5.7	<0.001	-4.060	134	-1.053
Potassium (meq/L)	4.59±1	4.04±1	0.013	2.121	134	0.550

Within the 2019 cohort, a comparison according to clinical outcome showed significant differences in several laboratory parameters. The mean hospital stay was 12±5 days in children with good outcome and 2±1 days in those with poor outcome (p<0.001), while mean ICU stay was 1±2 days and 1±1 day, respectively (p=0.583). Mean serum urea was 32±15 mg/dL in the good-outcome group compared with 58±38 mg/dL in the poor-outcome group (p=0.003), and mean serum creatinine was 0.7±0.3 mg/dL and 1.3±0.9 mg/dL, respectively (p=0.001). Mean pH was 7.135 in the good-outcome group and 6.904 in the poor-outcome group (p=0.011), while mean serum bicarbonate was 7.8 mmol/L and 5.2 mmol/L, respectively (p=0.098). Mean serum sodium was 134 mmol/L in the good-outcome group and 143 mmol/L in the poor-outcome group (p=0.004). Mean serum potassium was 4.68 mmol/L and 3.80 mmol/L, respectively (p=0.095), while mean HbA1c was 10.7% and 11.7%, respectively (p=0.406) (Table 5).

**Table 5:** Laboratory parameters according to outcome in the 2019 cohort ICU: Intensive Care Unit; pH: power of hydrogen; n: number of the study population; mg/dl: milligram per decilitre; meq/L: milliequivalent per litre; t: independent t-test; df: degree of freedom.

Variable	Good outcome (n=53)	Poor outcome (n=5)	p value	t value	df	Effect size (Cohen’s d)
Hospital stay (days)	12±5	2±1	<0.001	4.430	56	2.072
ICU stay (days)	1±2	1±1	0.583	0.000	56	0.000
Urea (mg/dL)	32±15	58±38	0.003	-3.146	56	-1.472
Creatinine (mg/dL)	0.7±0.3	1.3±0.9	0.001	-3.410	56	-1.595
pH	7.135±0.12	6.904±0.12	0.011	4.115	56	1.925
Bicarbonate (mg/dL)	7.8±3.6	5.2±3.6	0.098	1.544	56	0.722
Sodium (meq/L)	134±5.7	143±5.7	0.004	-3.375	56	-1.579
Potassium (meq/L)	4.68±1	3.8±1	0.095	1.881	56	0.880
HbA1c (%)	10.7±2.34	11.7±2.34	0.406	-0.913	56	-0.427

Among children in the 2021 cohort, a comparison according to COVID-19 IgG antibody status showed no statistically significant differences in the clinical characteristics, complications or outcomes assessed. Male sex was present in 14 (38.9%) children in the COVID IgG-positive group and 24 (57.1%) in the IgG-negative group (p=0.108). ICU admission was required in 25 (69.4%) and 35 (83.3%) children, respectively (p=0.147). Polyuria was reported in 22 (61.1%) children in the IgG-positive group and 28 (66.7%) in the IgG-negative group (p=0.610), while respiratory distress occurred in 21 (58.3%) and 21 (50.0%) children, respectively (p=0.462). Severe DKA was present in 17 (47.2%) and 18 (42.9%) children, respectively (p=0.475), and AKI occurred in 12 (33.3%) and 13 (31.0%) children, respectively (p=0.822). Poor outcome was observed in eight (22.2%) children in the IgG-positive group and four (9.5%) in the IgG-negative group (p=0.121). Sepsis was documented in 11 (30.6%) and 16 (39.0%) children, respectively (p=0.437) (Table 6).

**Table 6:** Comparison of clinical characteristics according to COVID-19 IgG antibody status ICU: Intensive Care Unit; AKI: acute kidney injury; n: number of the study population; DKA: diabetic ketoacidosis; φ: phi value; χ²: chi-square value; df: degrees of freedom.

Variable	COVID + (n=36)	COVID – (n=42)	p value	χ² value	df	Effect size (φ)
Male sex	14 (38.9%)	24 (57.1%)	0.108	2.585	1	0.182
ICU stay	25 (69.4%)	35 (83.3%)	0.147	2.106	1	0.164
Polyuria	22 (61.1%)	28 (66.7%)	0.610	0.260	1	0.058
Respiratory distress	21 (58.3%)	21 (50.0%)	0.462	0.542	1	0.083
Severe DKA	17 (47.2%)	18 (42.9%)	0.475	0.149	1	0.044
AKI	12 (33.3%)	13 (31.0%)	0.822	0.050	1	0.025
Poor outcome	8 (22.2%)	4 (9.5%)	0.121	2.401	1	0.175
Sepsis	11 (30.6%)	16 (38.1%)	0.437	0.487	1	0.079

Among children in the 2019 cohort, comparison according to clinical outcome showed significant differences in several clinical characteristics and complications. Severe DKA was present in 20 (37.7%) children with good outcome and five (100.0%) children with poor outcome (p=0.012). AKI occurred in 11 (20.8%) children with good outcome and four (80.0%) children with poor outcome (p=0.013). Mechanical ventilation was required in 3 (5.7%) children with good outcome and five (100.0%) children with poor outcome (p<0.001). Cerebral oedema was observed in eight (15.1%) children with good outcome and five (100.0%) children with poor outcome (p<0.001). Inotrope use was required in four (7.5%) children with good outcome and five (100.0%) children with poor outcome (p<0.001). Sepsis was documented in six (11.3%) children with good outcome and 3 (60.0%) children with poor outcome (p=0.023) (Table 7).

**Table 7:** Comparison of clinical characteristics according to outcome in the 2019 cohort AKI: Acute kidney injury; n: number of the study population; DKA: diabetic ketoacidosis; φ: phi value; df: degrees of freedom. ^a^Fischer's exact test.

Variable	Good outcome (n=53)	Poor outcome (n=5)	p value	Effect size (φ)
Severe DKA	20 (37.7%)	5 (100.0%)	0.012^a^	0.353
AKI	11 (20.8%)	4 (80.0%)	0.013^a^	0.380
Mechanical ventilation	3 (5.7%)	5 (100.0%)	<0.001^a^	0.768
Cerebral oedema	8 (15.1%)	5 (100.0%)	<0.001^a^	0.571
Inotrope use	4 (7.5%)	5 (100.0%)	<0.001^a^	0.717
Sepsis	6 (11.3%)	3 (60.0%)	0.023^a^	0.377

Among children in the 2021 cohort, comparison according to clinical outcome showed significant differences in several clinical characteristics and complications. ICU admission was required in 48 (72.7%) children with good outcome and 12 (100%) children with poor outcome (p=0.039). Respiratory distress was present in 30 (45.5%) children with good outcome and 12 (100%) children with poor outcome (p<0.001). Severe DKA was observed in 25 (37.9%) children with good outcome and 10 (83.3%) children with poor outcome (p=0.013). AKI occurred in 17 (25.8%) children with good outcome and eight (66.7%) children with poor outcome (p=0.005). Mechanical ventilation was required in four (6.1%) children with good outcome and 11 (91.7%) children with poor outcome (p<0.001). Cerebral oedema was present in 14 (21.2%) children with good outcome and 10 (83.3%) children with poor outcome (p<0.001). Inotropic support was required in six (9.1%) children with good outcome and 10 (83.3%) children with poor outcome (p<0.001). New-onset diabetes was observed in 40 (60.6%) children with good outcome and 11 (91.7%) children with poor outcome (p=0.037). Sepsis was documented in 18 (27.7%) children with good outcome and nine (75.0%) children with poor outcome (p=0.002) (Table 8).

**Table 8:** Comparison of clinical characteristics according to outcome in the 2021 cohort ICU: Intensive Care Unit; AKI: acute kidney injury; n: number of the study population; DKA: diabetic ketoacidosis; φ: phi value; χ²: chi-square value; df: degree of freedom.

Variable	Good outcome (n=66)	Poor outcome (n=12)	p value	χ² value	df	Effect size (φ)
ICU stay	48 (72.7%)	12 (100.0%)	0.039	4.255	1	0.234
Respiratory distress	30 (45.5%)	12 (100.0%)	<0.001	12.156	1	0.395
Severe DKA	25 (37.9%)	10 (83.3%)	0.013	8.481	1	0.330
AKI	17 (25.8%)	8 (66.7%)	0.005	7.803	1	0.316
Mechanical ventilation	4 (6.1%)	11 (91.7%)	<0.001	47.907	1	0.784
Cerebral oedema	14 (21.2%)	10 (83.3%)	<0.001	18.395	1	0.486
Inotrope use	6 (9.1%)	10 (83.3%)	<0.001	34.325	1	0.663
New-onset diabetes	40 (60.6%)	11 (91.7%)	0.037	4.328	1	0.236
Sepsis	18 (27.3%)	9 (75.0%)	0.002	10.219	1	0.362

In the overall study population, comparison according to clinical outcome showed significant differences in several clinical characteristics and complications. ICU admission was required in 82 (68.9%) children with good outcome and 16 (94.1%) children with poor outcome (p=0.030). Respiratory distress was present in 59 (49.6%) children with good outcome and 14 (82.4%) children with poor outcome (p=0.011). Severe DKA was observed in 45 (37.8%) children with good outcome and 15 (88.2%) children with poor outcome (p<0.001). AKI occurred in 28 (23.5%) children with good outcome and 12 (70.6%) children with poor outcome (p<0.001). Mechanical ventilation was required in seven (5.9%) children with good outcome and 16 (94.1%) children with poor outcome (p<0.001). Cerebral oedema was present in 22 (18.5%) children with good outcome and 15 (88.2%) children with poor outcome (p<0.001). Inotropic support was required in 10 (8.4%) children with good outcome and 15 (88.2%) children with poor outcome (p<0.001). Sepsis was documented in 24 (20.3%) children with good outcome and 12 (70.6%) children with poor outcome (p<0.001) (Table 9).

**Table 9:** Overall comparison of clinical characteristics according to outcome ICU: Intensive Care Unit; AKI: acute kidney injury; n: number of the study population; DKA: diabetic ketoacidosis; φ: phi value; χ²: chi-square value; df: degrees of freedom.

Variable	Good outcome (n=119)	Poor outcome (n=17)	p value	χ² value	df	Effect size (φ)
ICU stay	82 (68.9%)	16 (94.1%)	0.030	4.695	1	0.186
Respiratory distress	59 (49.6%)	14 (82.4%)	0.011	6.425	1	0.217
Severe DKA	45 (37.8%)	15 (88.2%)	<0.001	15.338	1	0.336
AKI	28 (23.5%)	12 (70.6%)	<0.001	15.867	1	0.342
Mechanical ventilation	7 (5.9%)	16 (94.1%)	<0.001	82.416	1	0.778
Cerebral oedema	22 (18.5%)	15 (88.2%)	<0.001	36.539	1	0.518
Inotrope use	10 (8.4%)	15 (88.2%)	<0.001	63.187	1	0.682
Sepsis	24 (20.2%)	12 (70.6%)	<0.001	19.429	1	0.378

## Discussion

The present study evaluated differences in the clinical profile, biochemical parameters, complications and outcomes of pediatric DKA before and during the COVID-19 pandemic. The findings indicate that although age distribution, duration of hospital stay and ICU stay were comparable between the two cohorts, children presenting during the pandemic had significantly higher HbA1c values and a significantly greater incidence of sepsis. In addition, there was a trend towards increased disease severity and complications during the COVID era. These observations are consistent with reports from several countries describing changes in the presentation of pediatric DKA during the pandemic [8].

Age at presentation was similar in both groups, suggesting that the pandemic did not substantially alter the demographic characteristics of children presenting with DKA. Similar observations have been reported by Kamrath et al., who found that the age distribution of children presenting with newly diagnosed type 1 diabetes remained comparable during the COVID-19 pandemic despite a significant increase in DKA and severe DKA [7]. Han and Heo also reported no significant difference in anthropometric characteristics between children presenting with DKA before and during the COVID-19 period [13].

A notable finding of the present study was the significantly higher HbA1c level among children admitted during the COVID period despite lower admission blood glucose levels. Elevated HbA1c reflects poorer long-term glycaemic control and may indicate delayed diagnosis or disruption of routine diabetes care during the pandemic. Fredheim et al. demonstrated that DKA at the onset of type 1 diabetes was associated with poorer subsequent glycaemic control, supporting the importance of the metabolic state at diagnosis in determining subsequent diabetes management [14]. During the COVID-19 pandemic, reduced healthcare utilization, interruption of routine follow-up and delayed presentation may have contributed to poorer glycaemic control before diagnosis. Kamrath et al. similarly suggested that reduced healthcare utilization and delayed medical attention were important factors contributing to increased DKA during the pandemic [7].

The incidence of sepsis increased significantly during the pandemic in the present study. Infection is an important precipitating factor for DKA and has been associated with adverse outcomes in children with severe metabolic decompensation. A South Indian study by Poovazhagi identified infection among the important precipitating factors associated with pediatric DKA and emphasized its contribution to clinical deterioration [11]. Jayashree and Singhi similarly reported sepsis and septic shock as important causes of mortality among children with DKA admitted to a pediatric intensive care unit [15]. The higher incidence of sepsis observed in our COVID-era cohort may therefore reflect greater systemic illness and delayed presentation rather than a direct effect of SARS-CoV-2 infection itself.

The subgroup analysis according to COVID-19 IgG antibody status demonstrated no significant differences in biochemical severity, complications or outcome between antibody-positive and antibody-negative children. This finding suggests that previous SARS-CoV-2 exposure, as reflected by antibody positivity, was not independently associated with the severity or short-term outcome of DKA in our cohort. This observation is consistent with the findings of studies suggesting that indirect effects of the pandemic, including delayed healthcare access and interruption of diabetes services, may have contributed substantially to the increased burden of DKA [7]. The Korean study by Han and Heo similarly concluded that the increased incidence of pediatric DKA during the pandemic could be related to indirect effects of the pandemic rather than demonstrable differences in disease severity or complications [13].

The present study identified renal dysfunction and severe metabolic acidosis as important biochemical predictors of adverse outcome. Children with poor outcome had significantly higher serum urea and creatinine concentrations together with markedly lower pH and bicarbonate values in both the pre-pandemic and pandemic cohorts. Jayashree et al. reported that children who died from DKA had more severe acidosis at admission and persistent metabolic abnormalities during treatment [15]. Poovazhagi also identified severe acidosis, renal dysfunction and other markers of systemic deterioration as important factors associated with mortality in children with DKA from developing countries [11].

Hypernatremia was another significant predictor of poor outcome in the present study. Elevated serum sodium may reflect severe dehydration and hyperosmolarity and may be associated with an increased risk of neurological complications. Careful monitoring of serum sodium and other electrolytes is therefore essential during the management of pediatric DKA. The importance of cerebral complications and metabolic derangements in determining outcome has also been emphasized in previous pediatric DKA studies [2].

The categorical analysis further demonstrated that severe DKA, acute kidney injury, cerebral oedema, respiratory distress, mechanical ventilation, inotrope requirement and sepsis were all strongly associated with adverse outcome. Similar findings have been reported by Jayashree and Singhi, who identified severe acidosis, increased osmolality, persistent metabolic abnormalities and complications such as cerebral oedema and septic shock among important determinants of mortality [15]. Studies from India have also demonstrated a strong association between severe DKA and cerebral oedema. Agarwal et al. found that cerebral oedema was particularly associated with severe metabolic acidosis, lower arterial carbon dioxide levels, prior fluid administration and new-onset diabetes [16]. Tiwari et al. similarly identified fluid-refractory shock and other markers of severe illness as important risk factors for cerebral oedema in children with DKA [17].

The strong association between cerebral oedema and poor outcome in the present study is also consistent with previous international evidence. Glaser et al. identified several clinical and biochemical risk factors associated with cerebral oedema in children with DKA and demonstrated its important relationship with morbidity and mortality [18]. These findings emphasize the need for early recognition of neurological deterioration and careful management of fluid and metabolic abnormalities in children with severe DKA.

The association between multiorgan dysfunction and adverse outcomes observed in our study is also supported by previous pediatric DKA literature. Syed et al. reported that severe metabolic abnormalities and complications were associated with poor outcomes among children with DKA in a tertiary care setting [19]. Similarly, Ganesh et al. described severe DKA and associated complications as important determinants of outcome in children with DKA [20]. The consistency of these findings across different populations supports the importance of early recognition of severe DKA and timely intensive care intervention.

Interestingly, new-onset diabetes was significantly more frequent among children with poor outcome during the pandemic. This finding is consistent with international reports describing an increase in new-onset type 1 diabetes presenting with DKA during the COVID-19 pandemic. Unsworth et al. [6] reported an increase in new-onset type 1 diabetes among children during the pandemic, while Kamrath et al. [7] demonstrated a significant increase in both DKA and severe DKA at diagnosis during the pandemic period. A subsequent meta-analysis involving more than 124,000 children with diabetes also demonstrated an increased risk of DKA, particularly severe DKA, among newly diagnosed children during the pandemic [8]. However, the association observed in our study should be interpreted cautiously because the number of children with poor outcome was relatively small.

Strengths of the study

The strengths of the present study include direct comparison of pre-pandemic and pandemic cohorts, evaluation of COVID-19 antibody status and comprehensive assessment of biochemical and clinical predictors of outcome. The study also provides regional data from a tertiary care centre in South India, where pediatric data regarding the impact of the COVID-19 pandemic on DKA remain relatively limited.

Limitations of the study

However, several limitations should be acknowledged. The single-centre design limits generalizability, while the relatively small subgroup sizes, particularly the number of children with poor outcome, reduce statistical power for subgroup comparisons. Because this was an observational comparison of pre-pandemic and pandemic periods, the observed differences cannot be attributed solely to direct SARS-CoV-2 infection. The findings may reflect a combination of factors, including changes in healthcare access, delayed presentation, lockdown-related disruption, changes in health-seeking behaviour and SARS-CoV-2 exposure. A major limitation of the study was the relatively small sample size, which was largely attributable to a significant reduction in patient referrals to the tertiary care center during the COVID-19 pandemic. This may have limited the statistical power and generalizability of the study findings. The relatively small number of poor-outcome events limited the ability to perform robust multivariable modelling without the potential for overfitting. Therefore, associations identified in univariate analyses should be interpreted cautiously and should not be considered evidence of independent causal effects. Potential differences in ICU admission thresholds, referral patterns and healthcare utilization during the pandemic may also have influenced the clinical profile of children admitted to intensive care in 2021 compared with 2019. This possibility may have introduced selection bias into comparisons involving ICU-related variables and outcomes. The study also used different data-collection approaches across the two periods, with retrospective chart-based data for 2019 and prospective data collection during 2021. Delayed presentation to hospital may have contributed to differences in DKA severity and outcomes during the pandemic period. However, the present study did not have sufficiently detailed and consistently recorded data on symptom-to-presentation intervals to reliably evaluate the interaction between delayed presentation and disease severity or to determine whether delayed presentation independently explained the observed differences between study periods. Differences in documentation completeness and ascertainment between the two approaches may have introduced information bias and should be considered when interpreting comparisons between the study periods. In addition, part of the dataset was collected retrospectively, which may have resulted in incomplete documentation of some clinical variables. The observational design also prevents establishing a causal relationship between the COVID-19 pandemic and changes in DKA severity. Finally, COVID-19 IgG positivity indicates previous exposure but does not establish the timing or clinical severity of SARS-CoV-2 infection.

## Conclusions

The present study demonstrated differences in selected clinical and biochemical characteristics of children presenting with DKA during the COVID-19 pandemic compared with the pre-pandemic period. During the pandemic period, higher HbA1c levels were observed, while blood glucose levels were lower compared with the pre-pandemic period. COVID-19 IgG status was not significantly associated with differences in the clinical characteristics, biochemical parameters or outcomes evaluated in the 2021 cohort. Poor outcomes were associated with greater severity of illness, including severe diabetic ketoacidosis, acute kidney injury, respiratory distress, mechanical ventilation, cerebral oedema, inotrope requirement and sepsis. These findings indicate an association between markers of acute illness severity and poor hospital outcomes rather than establishing independent or causal effects.

The findings should be interpreted in the context of the observational study design and the limited number of poor-outcome events. Differences between the pre-pandemic and pandemic periods may also have been influenced by delayed presentation, changes in healthcare access and health-seeking behaviour and differences in referral or intensive care admission practices. In addition, the use of retrospective data for 2019 and prospective data collection for 2021 may have introduced differences in documentation and ascertainment. Further prospective multicentre studies with larger sample sizes and standardized data collection are required to better evaluate the factors contributing to pediatric DKA severity and outcomes during periods of healthcare disruption.
